# Supplementary material for: The interactome of intact mitochondria by cross-linking mass spectrometry provides evidence for coexisting respiratory supercomplexes
Source: Mol Cell Proteomics. 2017 Dec 8;17(2):216–32. doi: 10.1074/mcp.RA117.000470 (PMC5795388; doi:10.1074/mcp.RA117.000470)
Supplement: Supplemental Data [file supp_RA117.000470_133922_0_supp_23978_fzffwf.zip › spectra_annotation/mito_spectra_annotation/391-1-6-1-4-1.pdf]

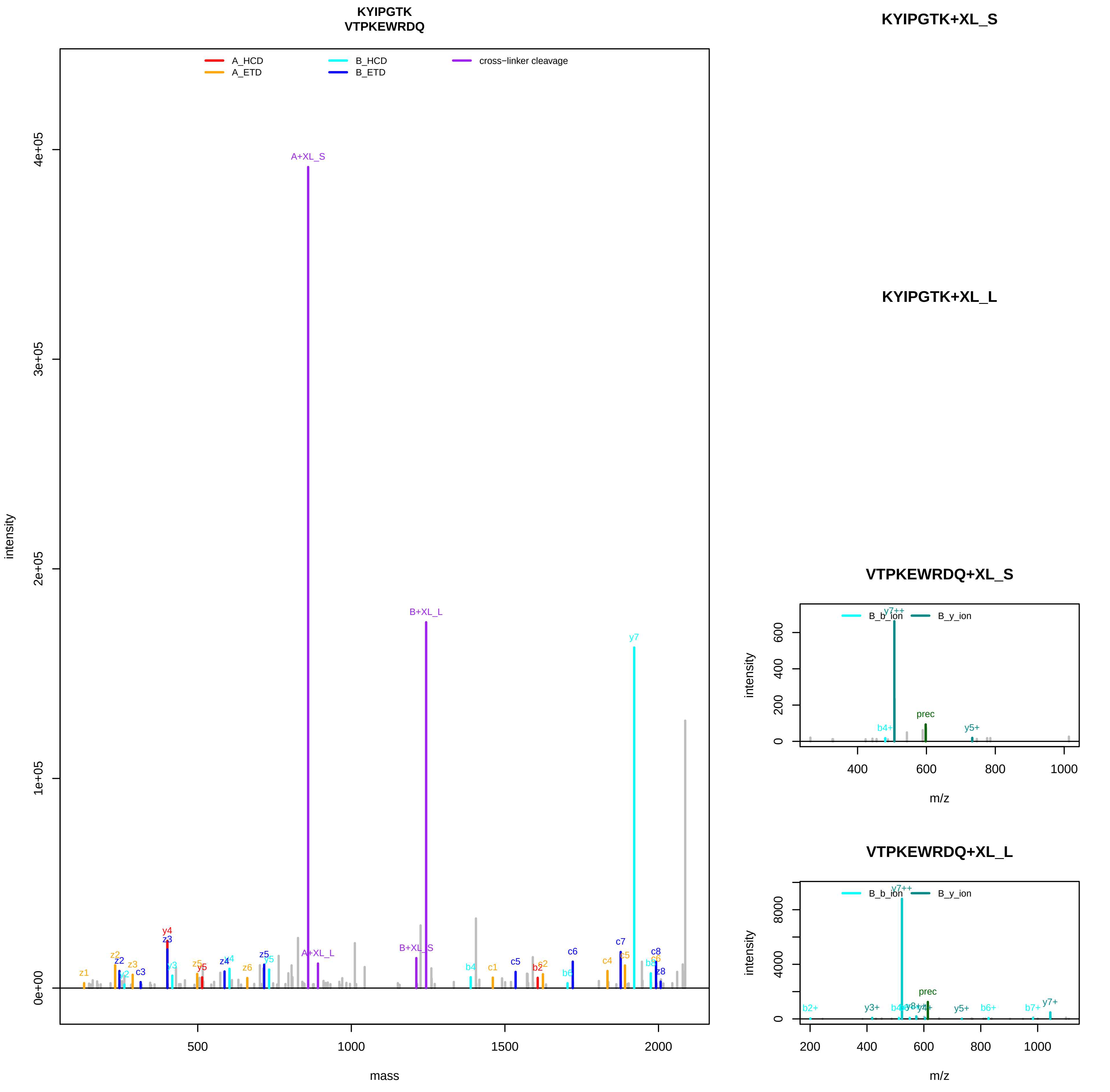
KYIPGTK
VTPKEWRDQ
A_HCD
A_ETD
B_HCD
B_ETD
cross−linker cleavage
intensity
mass
0e+00
1e+05
2e+05
3e+05
4e+05
500
1000
1500
2000
A+XL_S
B+XL_L
y7
A+XL_L
B+XL_S
z1
z2
z3
c3
y4
z3
y3
z5
y5
z4
y4
z6
z5
y5
b4
c1
c5
b2
c2
b6
c6
c4
c7
c5
b8
c6
c8
z8
KYIPGTK+XL_S
KYIPGTK+XL_L
VTPKEWRDQ+XL_S
B_b_ion
B_y_ion
y7++
b4+
prec
y5+
m/z
0
200
400
600
400
600
800
1000
VTPKEWRDQ+XL_L
0
4000
8000
200
400
600
800
1000
b2+
y3+
y5+
b6+
b7+
y7+
